# The longitudinal relationship between helicopter parenting and depressive symptoms among university students: the serial mediating roles of exercise behavior and physical self-esteem

**DOI:** 10.3389/fpsyg.2026.1834318

**Published:** 2026-06-08

**Authors:** Yuqing Wang, Geng Li, Zifu Shi, Yihan Zhang, Biqi Xia, Lizirui Huang, Changchang Huang

**Affiliations:** 1 Cognition and Human Behavior Key Laboratory of Hunan, School of Educational Science, Hunan Normal University, Changsha, China; 2 School of Psychology, Shanghai University of Sport, Shanghai, China; 3 College of Physical Education, Hunan Normal University, Changsha, China; 4 School of Psychology and Cognitive Science, East China Normal University, Shanghai, China; 5 Hunan Sany Polytechnic College, Changsha, China

**Keywords:** depressive symptoms, exercise behavior, helicopter parenting, physical self-esteem, university students

## Abstract

Helicopter parenting has been linked to adverse mental health outcomes, yet the longitudinal mechanisms through which it may contribute to depressive symptoms remain unclear; this study examined the prospective association between helicopter parenting and depressive symptoms among university students using a three-wave design and tested a serial mediation model involving exercise behavior and physical self-esteem. A sample of 491 Chinese university students (157 males; mean age = 18.73 years, SD = 0.84) completed the Helicopter Parenting Scale, Physical Activity Rating Scale, Physical Self-Esteem Scale, and Self-Rating Depression Scale across three waves spaced 6 months apart. The results indicated that helicopter parenting was positively associated with depressive symptoms, and both exercise behavior and physical self-esteem significantly mediated this relationship; importantly, the hypothesized serial indirect effect of helicopter parenting on depressive symptoms via exercise behavior and physical self-esteem was significant. These findings demonstrate a prospective association between helicopter parenting and depressive symptoms and highlight the mediating roles of exercise behavior and physical self-esteem, suggesting that helicopter parenting represents a potential target for future intervention research aimed at reducing depressive symptoms in university students.

## Introduction

Against the backdrop of rapid socioeconomic development in China, university students face a distinct set of psychosocial challenges as they transition from adolescence to adulthood, including interpersonal difficulties, environmental adaptation, academic pressure, and future planning (Pittman and Richmond, 2008). These stressors contribute to a notably high burden of depressive symptoms within this population; recent meta-analytic evidence indicates that approximately 20%–30% of Chinese university students experience clinically significant depressive symptoms (Wu et al., 2020). Importantly, the number of students suffering from elevated depressive symptoms far exceeds those who receive a formal clinical diagnosis. Given the high prevalence and potential adverse consequences of depressive symptoms among university students, a detailed investigation into its underlying mechanisms is both urgent and essential.

### Helicopter parenting and university students’ depressive symptoms

Although behavioral genetics research indicates that depressive symptoms have a moderate heritability, estimated at approximately 30%–40% (e.g., Polderman et al., 2015; Sullivan et al., 2000), the family environment–including parenting style–interacts with genetic predispositions to shape students’ mental health outcomes. A substantial body of research has established a robust link between parenting behaviors and depressive symptoms among university students (Stavrulaki et al., 2021). At the same time, Self-Determination Theory (SDT) provides a useful framework for understanding why certain parenting styles are more detrimental than others. SDT posits that the satisfaction of basic psychological needs for autonomy, competence, and relatedness is essential for psychological well-being, and that controlling environments undermine this process (Ryan and Deci, 2017). For instance, autonomy-supportive parenting–which encourages independence and minimizes control–has been found to protect against depressive symptoms (Van der Giessen et al., 2023). In contrast, controlling parenting (e.g., intrusive decision-making, autonomy restriction, and excessive monitoring) thwarts the development of autonomy and increases the risk of depressive symptoms (Li et al., 2015).

In recent years, “helicopter parenting” has gained attention as a prototypical form of overcontrolling parenting, referring to parents’ developmentally inappropriate and highly intrusive behaviors toward their emerging adult children (Padilla-Walker and Nelson, 2012; Kouros et al., 2017). Through behavioral control, autonomy restriction, and the reinforcement of an external locus of attribution (Padilla-Walker and Nelson, 2012; Kwon et al., 2016), such parenting not only hinders competence recognition and social adaptation but has also been positively associated with neuroticism (Odenweller et al., 2014) and higher rates of antidepressant use (LeMoyne and Buchanan, 2011). In line with this theoretical and empirical background, the present study proposes the following hypothesis: Higher levels of helicopter parenting at Time 1 would be positively associated with higher levels of depressive symptoms at Time 3 (Hypothesis 1).

#### Exercise behavior as a mediator

Against this backdrop of controlling parenting, diminished engagement in exercise behavior may represent a critical pathway through which helicopter parenting heightens the risk of depressive symptoms among university students. From the perspective of self-determination theory (SDT), helicopter parenting–characterized by excessive control, intrusion, and limited autonomy support–is likely to undermine emerging adults’ intrinsic motivation for physical activity (Hagger and Chatzisarantis, 2008; Teixeira et al., 2012). When parents override emerging adults’ autonomy and volition, exercise, which often requires self-initiation and personal enjoyment, may be particularly susceptible to neglect. Moreover, beyond undermining intrinsic motivation, helicopter parenting–through overprotection and substitutionary arrangements–directly constricts young adults’ opportunities to establish a sense of control via autonomous bodily exploration. From the perspective of embodied cognition (Varela et al., 2017), such deprivation of sensorimotor agency may impede the development of a stable perception of autonomous mastery, further suppressing exercise engagement. This dynamic is especially pronounced within the Chinese sociocultural context, where strong parental expectations for academic achievement translate into stringent control over children’s daily activities (Leung and Shek, 2018), thereby potentially shaping students’ daily routines and opportunities for autonomous physical activity. Nevertheless, empirical evidence suggests that parental autonomy support, rather than controlling behaviors, facilitates sustained exercise participation in older adolescents (Morrison et al., 2013).

Converging evidence consistently demonstrates that regular, moderate exercise behavior is significantly associated with lower levels of depressive symptoms (Ruby et al., 2011). Longitudinal data further confirm that chronically low physical activity constitutes an independent risk factor for depressive symptoms (Roshanaei-Moghaddam et al., 2009), whereas individuals who exercise more frequently exhibit markedly milder depressive symptoms (Gudmundsson et al., 2015). The antidepressant effects of exercise behavior extend beyond enhanced self-efficacy and positive affect (Zhang, 2025) to encompass elevated levels of neurotransmitters such as endorphins, serotonin, and dopamine (Zhang et al., 2025), as well as the buffering effect of social interaction inherent in many forms of physical activity against loneliness. Therefore, considering that helicopter parenting reduces exercise behavior (as argued above), and that reduced exercise increases depressive symptoms risk, the present study hypothesizes that exercise behavior mediates the relationship between helicopter parenting and depressive symptoms among university students (Hypothesis 2).

#### Physical self-esteem as a mediator

In the context of controlling parenting, the erosion of domain-specific self-esteem–particularly physical self-esteem–may further illuminate the pathway from helicopter parenting to depressive symptoms among university students. Self-determination theory (Ryan and Deci, 2017) provides a unifying framework for understanding this process: when parenting behaviors consistently thwart the basic psychological needs for autonomy and competence, individuals are likely to experience diminished self-worth across various self-domains. Helicopter parenting, characterized by intrusive monitoring and restriction of autonomous choices, directly deprives emerging adults of opportunities to develop a sense of mastery over their own bodies and actions. The frustration of autonomy and competence needs not only undermines global self-esteem but also specifically damages physical self-esteem–that is, the valuation of one’s physical capabilities, appearance, and bodily control. In an overcontrolling family environment, where children’s autonomous choices are systematically replaced by parental decisions, the lack of opportunities for self-initiated physical activities and bodily exploration leads to a reduced sense of physical competence and, consequently, lower physical self-esteem. This domain-specific decline in self-worth may, in turn, contribute to depressive symptoms, as the persistent frustration of basic psychological needs is well-documented to increase vulnerability to depressive symptoms (Ryan and Deci, 2017).

Physical self-esteem, defined as an individual’s perception and evaluation of their own appearance, body shape, and physical competence (von Soest et al., 2017), emerges early in the development of self-awareness and is highly susceptible to environmental feedback. Whereas autonomy-supportive parenting fosters positive physical self-esteem by affirming the individual’s sense of agency and competence (Deci and Ryan, 2000), the rigid control and conditional regard inherent in helicopter parenting undermine these very perceptions. Deprived of autonomous bodily experiences and exposed to persistent external evaluation, young adults may internalize a negative view of their physical selves, especially in the context of social comparison (Wilson et al., 2001).

Compromised physical self-esteem, in turn, constitutes a robust predictor of depressive symptomatology. Individuals with higher physical self-esteem are better equipped to seek social support and mobilize coping resources when confronting adversity, thereby mitigating the onset of depressive symptoms (Morken et al., 2018). Conversely, low physical self-esteem is associated with heightened psychological burden, poorer stress regulation, and the gradual accumulation of depressive affect (Davis and Katzman, 1997). By systematically eroding the foundations of physical self-worth, helicopter parenting may deprive university students of a critical psychological buffer against depressive symptoms. Accordingly, this study hypothesizes that physical self-esteem mediates the relationship between helicopter parenting and depressive symptoms among university students (Hypothesis 3).

#### Serial mediating roles of exercise behavior and physical self-esteem

A systematic review of quantitative studies on overparenting indicates that this parenting style is associated with a range of negative developmental outcomes among emerging adults across psychological, behavioral, and social domains (Cui et al., 2022). In the present study, we propose that exercise behavior and physical self-esteem may jointly function as serial mediators in the relationship between helicopter parenting and depressive symptoms among university students. From the perspective of self-determination theory (Ryan and Deci, 2017), helicopter parenting–characterized by excessive control and intrusive involvement–thwarts emerging adults’ basic psychological need for autonomy, thereby undermining both their intrinsic motivation to engage in self-initiated activities and their sense of self-worth. Recent research supports this mechanism: helicopter parenting has been shown to significantly diminish emerging adults’ autonomy, self-esteem, and self-efficacy, which in turn predicts increased anxiety and depressive symptoms (La Rosa et al., 2025). Moreover, helicopter parenting is directly associated with a reduced sense of confidence and an increased tendency to avoid new challenges or activities (La Rosa et al., 2024), which may contribute to decreased engagement in health-promoting behaviors.

Regarding exercise behavior specifically, emerging evidence suggests that helicopter parenting constrains young adults’ participation in physical activity. A recent study among Chinese university students found that parenting styles are significantly associated with exercise behavior (Zhao et al., 2024). Although research on helicopter parenting and physical activity remains mixed, studies have documented that over-involved parents frequently monitor and restrict their adult children’s health behaviors (Fingerman, 2017), and the autonomy-suppressing nature of helicopter parenting (Segrin et al., 2022) may indirectly reduce intrinsic motivation for exercise. Once engaged, however, exercise behavior fosters a sense of self-determined pleasure, which in turn enhances physical self-esteem (He and Ji, 2003).

Physical self-esteem has been established as a key mechanism linking helicopter parenting to depressive symptoms. A recent empirical study with Chinese university students demonstrated that helicopter parenting significantly negatively impacted physical self-esteem (β = −0.75, *p* < 0.001), which in turn mediated the relationship between helicopter parenting and depressive symptoms (Wang et al., 2024). More broadly, a scoping review of studies published between 2020 and 2024 confirmed that helicopter parenting is significantly associated with increased depressive symptoms among university students, with factors such as self-esteem and autonomy serving as mediators (La Rosa et al., 2025). Building on these findings and the previous discussion of exercise behavior as a mediator, we further propose a sequential mediation pathway. Specifically, this study hypothesizes a serial indirect association in which higher levels of helicopter parenting are associated with lower exercise engagement, which is in turn associated with lower physical self-esteem and greater depressive symptoms (Hypothesis 4).

Although some studies have explored the association between helicopter parenting and depressive symptoms among university students (Schønning and Kallesten, 2022), most have employed cross-sectional designs, leaving the causal relationship between the two constructs in need of further investigation. To date, empirical research on the underlying mechanisms of this relationship remains limited, particularly within the Chinese cultural context, where parental control tends to be more stringent and the phenomenon more pronounced.

**FIGURE 1:**
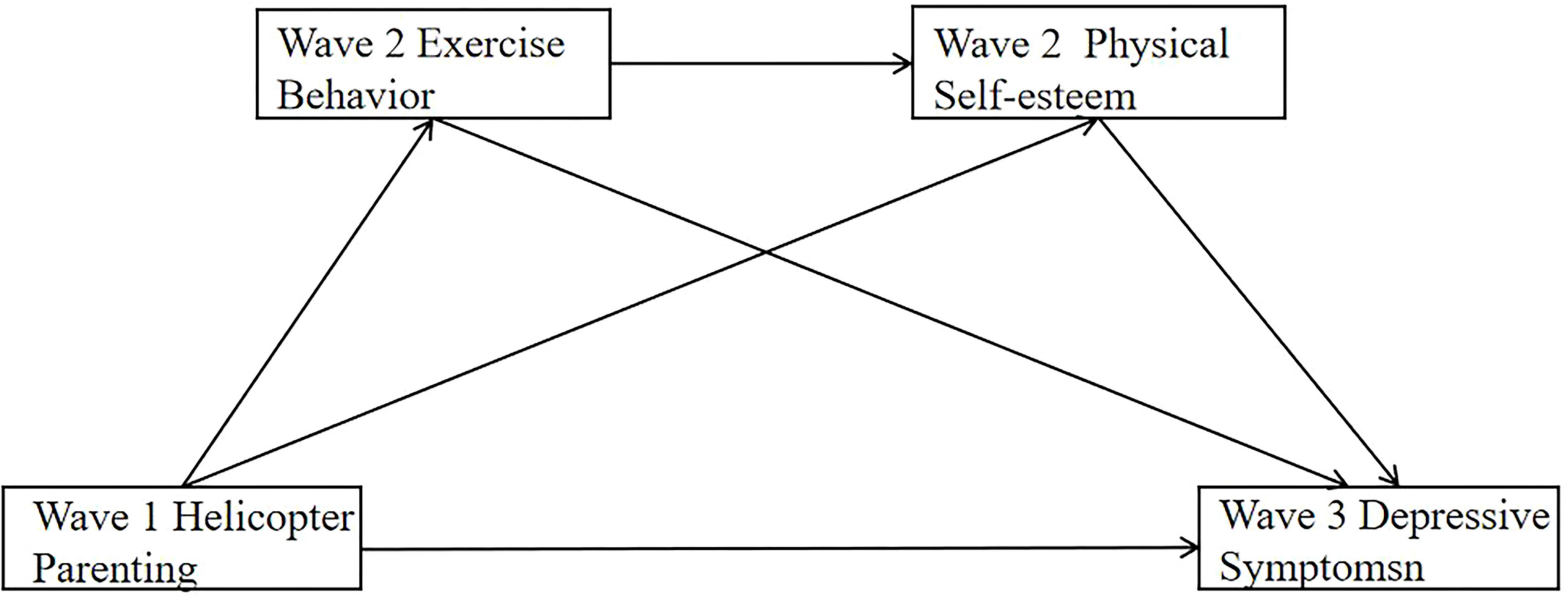
Hypothesized serial mediation model.

In summary, this study tests a serial mediation model (see Figure 1) to examine the longitudinal association between helicopter parenting and depressive symptoms among Chinese university students, as well as the mediating roles of exercise behavior and physical self-esteem in this association.

## Materials and methods

### Participants

This study was approved by the Institutional Review Board of the authors’ university. Data were collected online across three waves at 6-months intervals. Using a convenience sampling method, 551 university students (male = 176, female = 375; aged 17–22 years, *M* = 18.60, SD = 0.79) were recruited from five universities in Hunan, Jiangsu, Shanxi, and Guangdong provinces and completed the Wave 1 survey. At Wave 2, 523 participants (male = 172, female = 351; *M* = 18.81, SD = 0.73) completed the measures, and at Wave 3, 516 participants (male = 157, female = 359; *M* = 18.73, SD = 0.84) completed the measures. To assess potential attrition bias, participants were divided into an attrition group (those lost or excluded) and a retained group. A chi-square test showed no significant difference in gender distribution between the two groups, χ^2^ (1, *N* = 551) = 0.16, *p* = 0.691. Independent-samples *t*-tests indicated no significant differences in age, *t*(549) = −0.31, *p* = 0.757, or T1 helicopter parenting scores, *t*(549) = 0.88, *p* = 0.379. All *p*-values exceeded 0.05, indicating no selective attrition based on these baseline variables, which supports the interpretability and generalizability of the findings. For each participant, a completion rate was calculated (number of items actually answered ÷ total number of items × 100%), and participants with a completion rate below 80% were excluded (*n* = 8). For the remaining participants, missing values were imputed using the series mean method via the Replace Missing Values procedure in SPSS 24.0. The proportion of imputed missing values was 3%. Total scores were then calculated for each core scale, and cases where any scale total deviated from the overall mean by more than ±3 standard deviations were excluded as outliers (*n* = 17). The final valid sample consisted of 491 participants (male = 157, female = 334; retention rate: 89.1%).

### Measures

#### Helicopter parenting scale (HPS)

At Wave 1, we assessed helicopter parenting scale (HPS; Padilla-Walker and Nelson, 2012) The scale consists of 5 test items, for example, “My parents make crucial decisions for me, like where I should live, work, or the courses I should enroll in.” The scale uses a 5-point scoring system, with 1 being “completely disagree” and 5 being “completely agree.” A higher score indicates a higher level of helicopter parenting experienced by the university student. The internal consistency of the HPS items was excellent in our sample (α = 0.82).

#### Physical Activity Rating Scale-3 (PARS-3)

At Wave 2, participants’ exercise behavior was assessed using the Physical Activity Rating Scale-3 (PARS-3; Liang, 1994). The PARS-3 comprises three items that evaluate exercise intensity, duration (minutes per session), and frequency (times per month/week). Each item is rated on a 5-point Likert scale ranging from 1 to 5, with higher scores indicating greater engagement. Example items include: for intensity, options range from “1 = Light exercise (e.g., walking, radio calisthenics)” to “5 = Vigorous, sustained exercise with heavy breathing and sweating (e.g., racing, aerobics routines, swimming)”; for duration, options range from “1 = Less than 10 min” to “5 = Over 60 min”; and for frequency, options range from “1 = Less than once a month” to “5 = About once a day.” Following the established scoring protocol, an overall exercise behavior score is calculated using the formula: Exercise Behavior Score = Intensity × (Duration − 1) × Frequency. This composite score reflects the overall volume of physical activity, with possible values ranging from 0 to 100. In the present sample, the internal consistency reliability of the three items was good (α = 0.71).

#### Physical self-perception profile (PSPP)

At Wave 2, we assessed physical self-perception profile (PSPP; Xu and Yao, 2001). The scale consists of 30 test items, encompassing five dimensions: athletic competence, physical condition, body condition, self-worth, and physical attractiveness. For example, “I think it’s easy for me to keep my body attractive.” The scale uses a 4-point scoring system, with 1 being “strongly disagree” and 4 being “strongly agree.” A higher score indicates a higher level of physical self-esteem among university students. The internal consistency of the PSPP items was excellent in our sample (α = 0.93).

#### Self-Rating Depression Scale (SDS)

At Wave 3, The Self-Rating Depression Scale (SDS) was originally developed by Zung (1965) and later revised by Wu (1990) to assess depressive symptoms in China. The scale consists of 20 test items, covering four dimensions: psychological-affective symptoms, somatic disturbances, psychomotor disturbances, and psychological impediments of depressive symptoms. For example, “I feel useful and indispensable.” The scale uses a 4-point scoring system, with 1 being “strongly disagree” and 4 being “strongly agree.” A higher score indicates a higher frequency of depressive symptoms in the university student. The internal consistency of the SDS items was excellent in our sample (α = 0.90).

### Data analysis

Statistical analyses were conducted using SPSS 24.0 for descriptive statistics and correlation analyses on the collected valid data. Indirect effects were tested using 5,000 bootstrap samples with 95% bias-corrected confidence intervals. Gender was included as a control variable in the mediation analyses. Subsequently, hypothesis testing was performed using the PROCESS macro for SPSS (Model 6). In the analysis, gender is used as a control variable.

#### Controlling for and examining common method bias

To address potential common method bias, procedural remedies, including reverse-scoring of selected items, were implemented in the research design (Zhou and Long, 2004). Furthermore, Harman’s single-factor test was conducted on the collected data to statistically assess common method variance. The results revealed 13 factors with eigenvalues greater than 1, and the first factor accounted for 18.71% of the variance (below the 40% threshold). Thus, it can be concluded that no substantial common method bias was present in this study.

#### Descriptive statistics

Table 1 presents the descriptive statistics and correlation coefficients for the studied variables. The correlation analysis revealed that T1 helicopter parenting was significantly negatively correlated with both T2 exercise behavior and T2 physical self-esteem, but significantly positively correlated with T3 depressive symptoms. T2 exercise behavior was significantly positively correlated with T2 physical self-esteem and significantly negatively correlated with T3 depressive symptoms. A significant negative correlation was also observed between T2 physical self-esteem and T3 depressive symptoms.

**TABLE 1:** Correlation analysis between variables.

Variable	1	2	3	4
1 T1 helicopter parenting	1	1	1	1
2 T2 exercise behavior	−0.10*			
3 T2 physical self-esteem	−0.22***	0.25***		
4 T3 depressive symptoms	0.24***	−0.19***	−0.20***	
*M*	1.99	24.67	2.76	3.10
SD	1.46	17.71	2.36	1.92

T1 = time1, T2 = time2, T3 = time3,

***p < 0.001;

*p < 0.05; gender as a control variable.

#### Testing the serial mediation model

**FIGURE 2:**
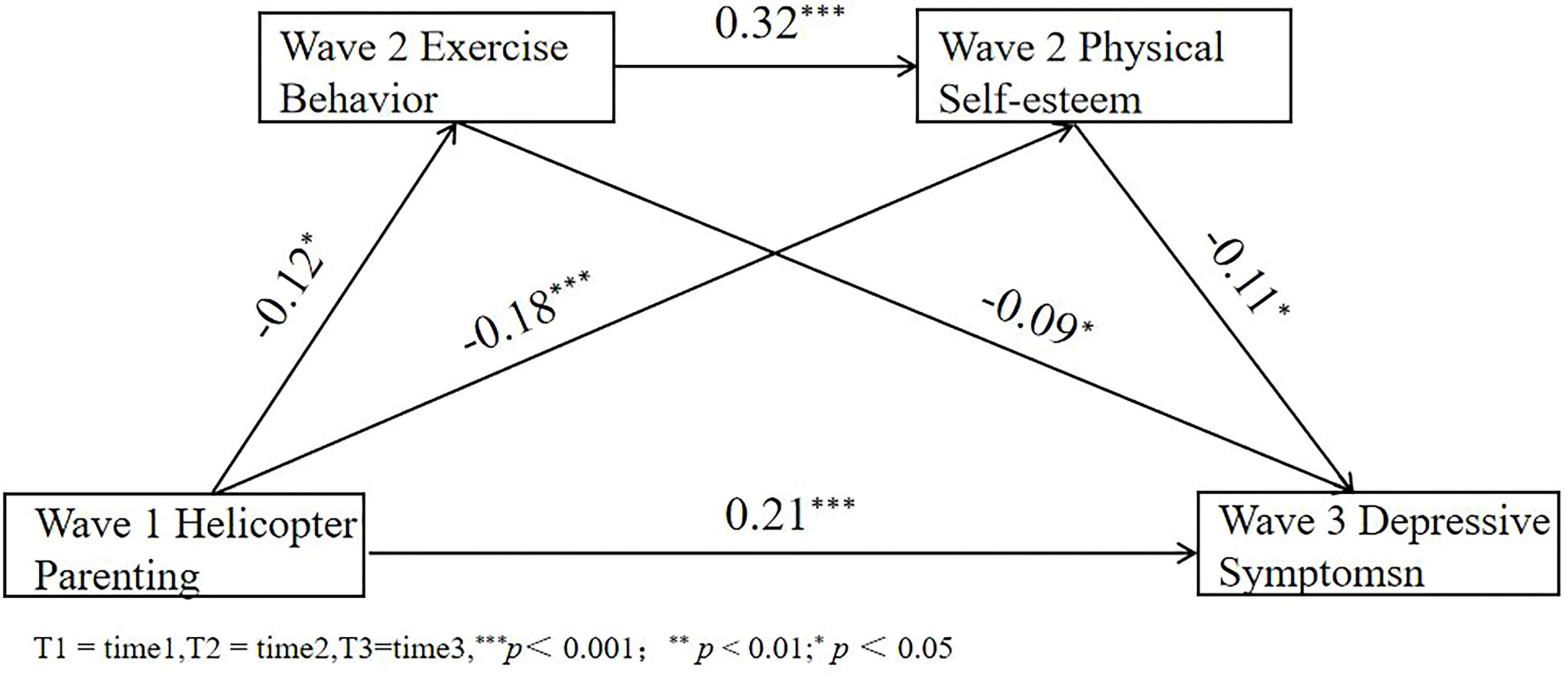
Serial mediation model with standardized path coefficients. T1 = time1, T2 = time2, T3 = time3, ***p < 0.001; **p < 0.01; *p < 0.05.

As shown in Figure 2 and Table 2, T1 helicopter parenting was significantly positively associated with T3 depressive symptoms (β = 0.213, *p* < 0.001). The indirect effect of T1 helicopter parenting on T3 depressive symptoms through T2 exercise behavior was significant (*B* = 0.0307, *SE* = 0.0214, 95% CI [0.0010, 0.0793]). The indirect effect through T2 physical self-esteem was also significant (*B* = 0.0646, *SE* = 0.0328, 95% CI [0.0083, 0.1376]). In addition, the serial indirect effect through T2 exercise behavior and T2 physical self-esteem was significant (*B* = 0.0140, *SE* = 0.0090, 95% CI [0.0008, 0.0349]).

**TABLE 2:** Direct and indirect effects in the serial mediation model.

Model pathways	*B*	*SE*	β	BC Boot 95% CI	
				Lower	Upper
Direct effect					
T1 helicopter parenting → T3 depressive symptoms	0.7043	0.1471	0.2129***	0.4153	0.9932
Indirect effects					
T1 helicopter parenting → T2 exercise behavior → T3 depressive symptoms	0.0307	0.0214	0.0093*	0.0010	0.0793
T1 helicopter parenting → T2 physical self-esteem → T3 depressive symptoms	0.0646	0.0328	0.0195**	0.0083	0.1376
T1 helicopter parenting → T2 exercise behavior → T2 physical self-esteem → T3 depressive symptoms	0.0140	0.0090	0.0042**	0.0008	0.0349

BC, bias-corrected; CI, confidence interval;

*p < 0.05;

**p < 0.01;

***p < 0.001.

## Discussion

The present study extends prior research on helicopter parenting and university students’ mental health in two respects. First, using a three-wave longitudinal design, it showed that perceived helicopter parenting at Time 1 was prospectively associated with depressive symptoms at Time 3. Second, it identified exercise behavior and physical self-esteem as two theoretically relevant indirect pathways, including a statistically significant serial indirect association consistent with the proposed model. Together, these findings suggest that the link between helicopter parenting and depressive symptoms may involve not only psychological self-evaluation but also health-related behavioral processes.

### Helicopter parenting and university students’ depressive symptoms

The primary objective of this study was to examine whether helicopter parenting is prospectively associated with depressive symptoms among Chinese university students and to explore potential mediating mechanisms. Consistent with Hypothesis 1, the results indicated that higher levels of helicopter parenting were significantly associated with elevated depressive symptoms. This finding aligns with a growing body of empirical evidence documenting the adverse mental health correlates of overparenting among emerging adults (Cui et al., 2019; La Rosa et al., 2025). From the perspective of self-determination theory (Ryan and Deci, 2017), this association may be understood as a consequence of thwarted basic psychological needs: helicopter parenting, characterized by excessive control and intrusive involvement, inherently undermines the development of autonomy and competence (Segrin et al., 2022). Prior research has established that diminished autonomy and heightened achievement-related anxiety are robust contributors to depressive symptomatology (Deci and Ryan, 2000; Nietzel and Harris, 1990). Thus, one plausible interpretation is that helicopter parenting may be linked to elevated depressive symptoms partly through its association with reduced opportunities for autonomous decision-making and self-directed accomplishment. Situated within the highly competitive Chinese educational context, where parental expectations and academic pressures are particularly salient (Leung and Shek, 2018), the present findings underscore the critical role of parenting practices in shaping university students’ psychological well-being. These findings further suggest that overcontrolling parenting may represent a meaningful target for future longitudinal and intervention research on depressive symptoms among university students.

#### Exercise behavior as a mediator

The results further demonstrated a significant indirect effect of helicopter parenting on depressive symptoms through exercise behavior, thereby supporting Hypothesis 2. Consistent with prior research documenting the inverse association between physical activity and depressive symptoms (Dishman et al., 2021), our findings indicate that higher levels of helicopter parenting were associated with lower levels of exercise engagement, which in turn was associated with elevated depressive symptoms. From the perspective of self-determination theory (Ryan and Deci, 2017), this pattern may reflect the autonomy-undermining nature of helicopter parenting: by imposing excessive control and restricting opportunities for self-initiated activity, helicopter parenting may be associated with weaker intrinsic motivation to engage in health-promoting behaviors such as exercise behavior (Segrin et al., 2022). Prior evidence further suggests that reduced physical activity may compromise neurobiological processes that support emotional regulation–for instance, by limiting the exercise-induced production of brain-derived neurotrophic factor (BDNF), a neurotrophin that is frequently diminished in individuals with depressive symptoms (Brunoni et al., 2008; Walsh and Tschakovsky, 2018). Although the present data do not permit direct causal conclusions regarding these physiological mechanisms, it is plausible to speculate that helicopter parenting may coincide with reduced engagement in exercise behavior, thereby limiting access to psychological and biological resources that are often associated with better emotional adjustment. Importantly, the current findings underscore that helicopter parenting is associated with depressive symptoms not solely through direct psychological pathways, but also through its potential to shape health-related behaviors in emerging adulthood.

#### Physical self-esteem as a mediator

The results also revealed a significant indirect effect of helicopter parenting on depressive symptoms via physical self-esteem, thereby confirming Hypothesis 3. Specifically, higher levels of helicopter parenting were associated with lower physical self-esteem, which in turn was associated with higher levels of depressive symptoms. The indirect effect was positive. This finding aligns with prior evidence linking physical self-esteem to depressive symptoms (Yuan et al., 2025; Orth et al., 2016). Within the self-determination theory framework, helicopter parenting may limit emerging adults’ opportunities to develop and affirm their physical competence and bodily agency, which may in turn be associated with lower physical self-esteem (Ryan and Deci, 2017). When emerging adults internalize a negative evaluation of their physical selves, they may become more vulnerable to depressive symptomatology. Although the precise mechanisms linking helicopter parenting to physical self-esteem warrant further investigation, one possibility is that overcontrolling parenting fosters an excessive reliance on external validation and social comparison regarding appearance and physical ability (Arslan et al., 2023), which may in turn amplify self-critical tendencies and undermine physical self-worth. The present findings therefore highlight a theoretically meaningful indirect pathway: higher perceived helicopter parenting was associated with lower physical self-esteem, which was in turn associated with greater depressive symptoms. This pattern is consistent with the possibility that autonomy-suppressing parenting experiences may be relevant to the development of poorer physical self-evaluation among emerging adults.

#### Serial mediating roles of exercise behavior and physical self-esteem

Leveraging the three-wave longitudinal design, our results supported a significant longitudinal serial indirect association linking T1 helicopter parenting with T3 depressive symptoms via T2 exercise behavior and T2 physical self-esteem, consistent with Hypothesis 4. Specifically, higher levels of helicopter parenting at Time 1 were associated with lower exercise behavior at Time 2; lower exercise behavior at Time 2 was associated with lower physical self-esteem at Time 2; and lower physical self-esteem at Time 2 was associated with higher depressive symptoms at Time 3. The serial indirect effect was positive. Because exercise behavior and physical self-esteem were assessed at the same wave, the ordering between these two mediators should be interpreted as theoretically specified rather than as evidence of temporal causality between them. From the perspective of self-determination theory, helicopter parenting may undermine autonomy and competence, thereby reducing students’ motivation to engage in self-initiated activities such as exercise behavior and weakening their sense of physical competence. These findings are consistent with the possibility that behavioral engagement and domain-specific self-evaluation jointly characterize one pathway linking overcontrolling parenting with depressive symptoms in university students.

## Limitations and future directions

Several limitations of the present study warrant acknowledgment. First, the sample was recruited via convenience sampling from only five universities across four provinces in China, which constrains the generalizability of the findings to the broader population of Chinese university students. Future research would benefit from adopting stratified random sampling or expanding geographic coverage to enhance representativeness. Second, the assessment of helicopter parenting did not differentiate between paternal and maternal influences, instead treating the construct as an overall parenting dimension. Given prior evidence suggesting that maternal helicopter parenting may exert less negative impact than paternal practices (Love et al., 2020), future studies should examine the distinct contributions of father-specific and mother-specific helicopter parenting to depressive symptoms. Third, all measures relied on self-report instruments, which may introduce reporting biases–particularly for exercise behavior, where subjective recall of frequency, duration, and intensity is prone to inaccuracy. Subsequent investigations could employ objective assessment tools, such as accelerometers or heart rate monitors, to obtain more precise physical activity data. Fourth, although this study identified exercise behavior and physical self-esteem as significant mediators, other psychological and physiological variables–including self-efficacy, self-awareness, and objective physical health indicators–may also function as underlying mechanisms linking helicopter parenting to depressive symptoms and merit further exploration. Fifth, although Harman’s single-factor test did not indicate substantial common-method bias (Podsakoff et al., 2003), all variables were assessed via self-report. Therefore, common-method variance and reporting bias cannot be fully ruled out. Future studies may benefit from incorporating multi-informant reports or objective measures, particularly for exercise behavior. Because depressive symptoms were assessed only at Time 3 and baseline depressive symptoms were not controlled, the present findings should be interpreted as longitudinal associations rather than evidence that helicopter parenting predicts changes in depressive symptoms over time. Future studies should include depressive symptoms at baseline and test cross-lagged or latent growth models to clarify directionality more rigorously. Moreover, there may be a bidirectional relationship between helicopter parenting and adolescent depressive symptoms: depressive symptoms may lead to overcontrolling parenting, while helicopter parenting may exacerbate depressive symptoms by undermining autonomy. Since the current study did not measure baseline depressive symptoms or examine reciprocal paths, the direction of causality cannot be determined. Therefore, future research should adopt multi-time-point longitudinal designs (e.g., cross-lagged or growth models) to clarify directionality and rule out confounding variables such as temperament and family climate.

## Conclusion

Based on a three-wave longitudinal survey of 491 university students, this study found that helicopter parenting was positively associated with depressive symptoms and negatively associated with exercise behavior and physical self-esteem. Exercise behavior and physical self-esteem each showed significant indirect effects in this association, and a significant serial indirect association consistent with the proposed model was also observed. These findings suggest that lower exercise engagement and reduced physical self-esteem may be important processes through which helicopter parenting is associated with depressive symptoms among university students. Future research and intervention studies may further examine whether reducing helicopter parenting and promoting exercise behavior and physical self-esteem can help alleviate depressive symptoms in this population.

## Data Availability

The original contributions presented in this study are included in the article/supplementary material, further inquiries can be directed to the corresponding authors.

## References

[B1] Arslan I. B. Lucassen N. Keijsers L. Stevens G. W. J. M. (2023). When too much help is of no help: Mothers’ and fathers’ perceived overprotective behavior and (mal)adaptive functioning in adolescents. *J. Youth Adolesc.* 52 1010–1023. 10.1007/s10964-022-01723-0 36633796 PMC10027782

[B2] Brunoni A. R. Lopes M. Fregni F. (2008). A systematic review and meta-analysis of clinical studies on major depression and BDNF levels: Implications for the role of neuroplasticity in depression. *Int. J. Neuropsychopharmacol.* 11 1169–1180. 10.1017/S1461145708009309 18752720

[B3] Cui M. Allen J. W. Fincham F. D. et al. (2019). Helicopter parenting, self-regulatory processes, and alcohol use among female college students. *J. Adult Dev.* 26 97–104. 10.1007/s10804-018-9301-5

[B4] Cui M. Hong P. Jiao C. (2022). Overparenting and emerging adult development: A systematic review. *Emerg. Adulth.* 10 1076–1094. 10.1177/21676968221108828

[B5] Davis C. Katzman M. (1997). Charting new territory: Body esteem, weight satisfaction, depression, and self-esteem among Chinese males and females in Hong Kong. *Sex Roles* 36 449–459. 10.1007/BF02766683

[B6] Deci E. L. Ryan R. M. (2000). The “what” and “why” of goal pursuits: Human needs and the self-determination of behavior. *Psychol. Inquiry* 11 227–268. 10.1207/S15327965PLI1104_01

[B7] Dishman R. K. McDowell C. P. Herring M. P. (2021). Customary physical activity and odds of depression: A systematic review and meta-analysis of 111 prospective cohort studies. *Br. J. Sports Med.* 55 926–934. 10.1136/bjsports-2020-103140 33402345

[B8] Fingerman K. L. (2017). Millennials and their parents: Implications of the new young adulthood for midlife adults. *Innov. Aging* 1:igx026. 10.1093/geroni/igx026 29795793 PMC5954613

[B9] Gudmundsson P. Lindwall M. Gustafson D. R. Östling S. Hällström T. Waern M. et al. (2015). Longitudinal associations between physical activity and depression scores in Swedish women followed 32 years. *Acta Psychiatr. Scand.* 132 451–458. 10.1111/acps.12419 25865488 PMC4600636

[B10] Hagger M. Chatzisarantis N. (2008). Self-determination theory and the psychology of exercise. *Int. Rev. Sport Exerc. Psychol.* 1 79–103. 10.1080/17509840701827437

[B11] He Y. Ji L. (2003). The effects of exercise behavior duration on depressive level and physical self-esteem in college students and verification of the mediation model. *Sports Sci.* 4 58–66.

[B12] Kouros C. D. Pruitt M. M. Ekas N. V. Kiriaki R. Sunderland M. (2017). Helicopter parenting, autonomy support, and college students’ mental health and well-being: The moderating role of sex and ethnicity. *J. Child Family Stud.* 26 939–949. 10.1007/s10826-016-0614-3 31832009 PMC6907082

[B13] Kwon K. A. Yoo G. Bingham G. E. (2016). Helicopter parenting in emerging adulthood: Support or barrier for Korean college students’ psychological adjustment? *J. Child Fam. Stud.* 25 136–145. 10.1007/s10826-015-0195-6

[B14] La Rosa V. L. Ching B. H.-H. Commodari E. (2024). The impact of helicopter parenting on emerging adults’ confidence and avoidance of new challenges. *J. Adult Dev.* 31 389–401.

[B15] La Rosa V. L. Ching B. H.-H. Commodari E. (2025). The impact of helicopter parenting on emerging adults in higher education: A scoping review of psychological adjustment in university students. *J. Genetic Psychol.* 186 162–189. 10.1080/00221325.2024.2413490 39757795

[B16] LeMoyne T. Buchanan T. (2011). Does “hovering” matter? Helicopter parenting and its effect on well-being. *Sociol. Spectr.* 31 399–418. 10.1080/02732173.2011.574038

[B17] Leung J. T. Y. Shek D. T. L. (2018). Validation of the perceived Chinese overparenting scale in emerging adults in Hong Kong. *J. Child Fam. Stud.* 27 103–117. 10.1007/s10826-017-0880-8

[B18] Li J. B. Delvecchio E. Lis A. (2015). Parental attachment, self-control, and depressive symptoms in Chinese and Italian adolescents: Test of a mediation model. *J. Adolesc.* 43 159–170. 10.1016/j.adolescence.2015.06.006 26132371

[B19] Liang D. Q. (1994). The stress level of college students and its relationship with exercise behavior. *Chinese J. Mental Health* 8 5–6.

[B20] Love H. Cui M. Allen J. Fincham F. D. May R. W. (2020). Helicopter parenting and female university students’ anxiety: Does parents’ gender matter? *Families Relationsh. Soc.* 9 417–430. 10.1332/204674319X15653625640669

[B21] Morken I. S. Røysamb E. Nilsen W. Karevold E. B. (2018). Body dissatisfaction and depressive symptoms on the threshold to adolescence: Examining gender differences in depressive symptoms and the impact of social support. *J. Early Adolesc.* 38 1237–1256. 10.1177/0272431618791280

[B22] Morrison S. A. Dashiff C. J. Vance D. E. (2013). Role of parental autonomy support on self-determination in influencing diet and exercise motivation in older adolescents. *Nurs. Res. Rev.* 3 77–85. 10.2147/NRR.S43795

[B23] Nietzel M. T. Harris M. J. (1990). Relationship of dependency and achievement/autonomy to depression. *Clin. Psychol. Rev.* 10 279–297. 10.1016/0272-7358(90)90063-G

[B24] Odenweller K. G. Booth-Butterfield M. Weber K. (2014). Investigating helicopter parenting, family environments, and relational outcomes for millennials. *Commun. Stud.* 65 407–425. 10.1080/10510974.2013.811434

[B25] Orth U. Robins R. W. Meier L. L. Conger R. D. (2016). Refining the vulnerability model of low self-esteem and depression: Disentangling the effects of genuine self-esteem and narcissism. *J. Pers. Soc. Psychol.* 110 133–149. 10.1037/pspp0000038 25915133

[B26] Padilla-Walker L. M. Nelson L. J. (2012). Black hawk down?: Establishing helicopter parenting as a distinct construct from other forms of parental control during emerging adulthood. *J. Adolesc.* 35 1177–1190. 10.1016/j.adolescence.2012.03.007 22503075

[B27] Pittman L. D. Richmond A. (2008). University belonging, friendship quality, and psychological adjustment during the transition to college. *J. Exp. Educ.* 76 343–362. 10.3200/JEXE.76.4.343-362

[B28] Podsakoff P. M. MacKenzie S. B. Lee J.-Y. Podsakoff N. P. (2003). Common method biases in behavioral research: A critical review of the literature and recommended remedies. *J. Appl. Psychol.* 88 879–903. 10.1037/0021-9010.88.5.879 14516251

[B29] Polderman T. J. C. Benyamin B. de Leeuw C. A. Sullivan P. F. van Bochoven A. Visscher P. M. et al. (2015). Meta-analysis of the heritability of human traits based on fifty years of twin studies. *Nat. Genet.* 47 702–709. 10.1038/ng.3285 25985137

[B30] Roshanaei-Moghaddam B. Katon W. J. Russo J. (2009). The longitudinal effects of depression on physical activity. *General Hosp. Psychiatry* 31 306–315. 10.1016/j.genhosppsych.2009.04.002 19555789

[B31] Ruby M. B. Dunn E. W. Perrino A. (2011). The invisible benefits of exercise. *Health Psychol.* 30 67–74. 10.1037/a0021859 21299296

[B32] Ryan R. M. Deci E. L. (2017). *Self-Determination Theory: Basic Psychological Needs in Motivation, Development, and Wellness.* New York, NY: Guilford Press, 10.1521/978.14625/28806

[B33] Schønning J. V. Kallesten K. B. (2022). A systematic review of “helicopter parenting” and its relationship with anxiety and depression. *Front. Psychol.* 13:872981. 10.3389/fpsyg.2022.872981 35693486 PMC9176408

[B34] Segrin C. Jiao J. Wang J. (2022). Indirect effects of overparenting and family communication patterns on mental health of emerging adults in China and the United States. *J. Adult Dev.* 29 205–217. 10.1007/s10804-022-09397-5

[B35] Stavrulaki E. Li M. Gupta J. (2021). Perceived parenting styles, academic achievement, and life satisfaction of college students: The mediating role of motivation orientation. *Eur. J. Psychol. Educ.* 36 693–717. 10.1007/s10212-020-00493-2

[B36] Sullivan P. F. Neale M. C. Kendler K. S. (2000). Genetic epidemiology of major depression: Review and meta-analysis. *Am. J. Psychiatry* 157 1552–1562. 10.1176/appi.ajp.157.10.1552 11007705

[B37] Teixeira P. J. Carraça E. V. Markland D. Silva M. N. Ryan R. M. (2012). Exercise, physical activity, and self-determination theory: A systematic review. *Int. J. Behav. Nutr. Phys. Activity* 9:78. 10.1186/1479-5868-9-78 22726453 PMC3441783

[B38] Van der Giessen D. de Rooij M. Meeus W. (2023). Longitudinal association between maternal autonomy support and controlling parenting and adolescents’ depressive symptoms. *J. Youth Adolesc.* 52 1058–1073. 10.1007/s10964-022-01722-1 36656443 PMC9851735

[B39] Varela F. J. Thompson E. Rosch E. (2017). *The Embodied Mind: Cognitive Science and Human Experience (Revised edition).* Cambridge, MA: MIT Press.

[B40] von Soest T. Wichstrøm L. Kvalem I. L. (2017). The development of global and domain-specific self-esteem from age 13 to 31. *Dev. Psychol.* 53 285–299. 10.1037/pspp0000060 26167796

[B41] Walsh J. J. Tschakovsky M. E. (2018). Exercise and circulating BDNF: Mechanisms of release and implications for the design of exercise interventions. *Appl. Physiol. Nutr. Metab.* 43 1095–1104. 10.1139/apnm-2018-0192 29775542

[B42] Wang C. Shi H. Li G. (2024). Helicopter parenting and college student depression: The mediating effect of physical self-esteem. *Front. Psychiatry* 14:1329248. 10.3389/fpsyt.2023.1329248 38264635 PMC10803400

[B43] Wilson P. M. Rodgers W. M. Fraser S. N. (2001). The relationship between exercise motives and physical self-esteem in female exercise participants: An application of Self-Determination Theory. *J. Appl. Biobehav. Res.* 6 79–104.

[B44] Wu D. Yu L. Yang T. Cottrell R. Peng S. Guo W. et al. (2020). The impacts of uncertainty stress on mental disorders of Chinese college students: Evidence from a nationwide study. *Front. Psychol.* 11:243. 10.3389/fpsyg.2020.00243 32210868 PMC7075936

[B45] Wu W. Y. (1990). Self-Rating Depression Scale (SDS). *Shanghai Arch. Psychiatry* 2 41–42.

[B46] Xu X. Yao J. X. (2001). Revision and test of college students’ physical self-esteem scale. *Sports Sci.* 21 78–81.

[B47] Yuan Y. Tu Y. Su Y. Jin L. Tian Y. Chang X. et al. (2025). The mediating effect of self-efficacy and physical activity with the moderating effect of social support on the relationship between negative body image and depression among Chinese college students: A cross-sectional study. *BMC Public Health* 25:285. 10.1186/s12889-025-21350-1 39849422 PMC11756201

[B48] Zhang S. (2025). The effect of exercise behavior on Chinese college students’ mental sub-health: The mediating role of mental resilience and the moderating role of self-efficacy. *Front. Psychol.* 16:1572974. 10.3389/fpsyg.2025.1572974 40625440 PMC12231497

[B49] Zhang Y. He J. Ruan H. Ma G. (2025). The impact of exercise behavior on mental health and the relationship among physical exercise, emotional regulation and suicidal ideation in Chinese medical students. *Front. Psychol.* 16:1609415. 10.3389/fpsyg.2025.1609415 40873524 PMC12379471

[B50] Zhao R. Yu J. Guo J. Wang X. (2024). Correlation between exercise behavior and parenting styles, and psychological resilience of college students. *Chinese J. School Health* 45 1152–1156. 10.16835/j.cnki.1000-9817.2024240

[B51] Zhou H. Long L. R. (2004). Gongtong fangfa piancha de tongji jianyan yu kongzhi fangfa [Statistical remedies for common method biases]. *Adv. Psychol. Sci.* 12 942–950.

[B52] Zung W. W. K. (1965). A self-rating depression scale. *Arch. General Psychiatry* 12 63–70. 10.1001/archpsyc.1965.01720310065008 14221692

